# Identification of 16,25-*O*-diacetyl-cucurbitane F and 25-*O*-acetyl-23,24-dihydrocucurbitacin F as novel anti-cancer chemicals

**DOI:** 10.1098/rsos.180723

**Published:** 2018-08-15

**Authors:** Wenxue Wang, Haoran Yang, Ying Li, Zhongfei Zheng, Yongjun Liu, Haiyang Wang, Yanling Mu, Qingqiang Yao

**Affiliations:** 1 School of Medicine and Life Sciences, University of Jinan-Shandong Academy of Medical Sciences, Jinan 250200, Shandong, People's Republic of China; 2 Institute of Materia Medica, Shandong Academy of Medical Sciences, Jinan 250062, Shandong, People's Republic of China; 3 Key Laboratory for Biotech-Drugs Ministry of Health, Jinan 250062, Shandong, People's Republic of China; 4 Key Laboratory for Rare and Uncommon Diseases of Shandong Province, Jinan 250062, Shandong, People's Republic of China

**Keywords:** Cucurbitaceae, *Hemsleya pengxianensis*, cucurbitane triterpenoid, anti-cancer

## Abstract

Seven new cucurbitane glucosides, hemslepensides J-P (**1**–**7**), and two known compounds, 16,25-*O*-diacetyl-cucurbitane F (**8**) and 25-*O*-acetyl-23,24-dihydrocucurbitacin F (**9**), were isolated from the tubers of *Hemsleya pengxianensis* var. *jinfushanensis*. The structures of **1**–**7** were elucidated using infrared absorption spectroscopy, nuclear magnetic resonance spectroscopy and high-resolution electrospray ionization mass spectrometry. The treatment of HT29 cells, human colon cancer cells, with compounds **8** and **9** inhibited cell proliferation. Further study demonstrated that compounds **8** and **9** induced F-actin aggregation, G_2_/M phase cell cycle arrest and cell apoptosis in HT29 cells. In summary, the present study enriched the chemical composition research of *H. pengxianensis*, and suggested that the compounds **8**/**9** treatment may be a potentially useful therapeutic option for colon cancer.

## 1. Introduction

Tetracyclic triterpenes are a diverse group of natural products consisting of four rings and 30 carbon atoms. Extracted from the rhizomes, roots or the juice of various plants such as *Siraitia grosvenorii* (Cucurbitaceae) or *Momordica* Linn. (Cucurbitaceae), tetracyclic triterpenes have been reported with multiple biological activities including anti-inflammatory function, cytotoxicity and anti-cancer, preventive and curative effects against CCl_4_-induced hepatotoxicity and anti-fertility activities [1–3]. The genus *Hemsleya* (Cucurbitaceae) includes approximately 30 species, distributed in the subtropical to temperate regions in Asia, eastern India and northern Vietnam. In China, it is mainly distributed in the southwest to south central regions. Tetracyclic triterpenes were identified as the main chemical constituents in the tubers of *Hemsleya pengxianensis* var. *jinfushanensis* [4–8].

**Figure 1.**
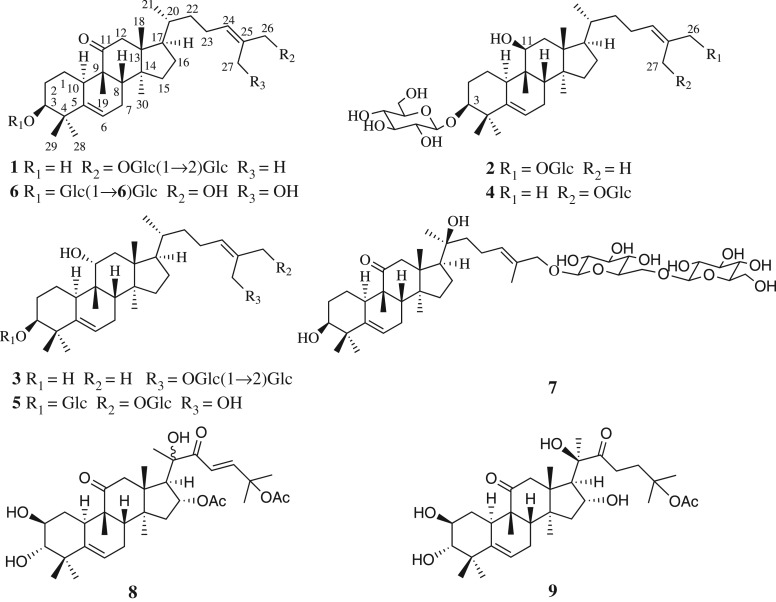
The structures of compounds **1**–**9.**

As previously reported, our studies had identified 16 new compounds with cytotoxic activity against the human cancer cell lines [6–8]. Recently, further investigation of *H. pengxianensis* var. *jinfushanensis* resulted in the discovery of another seven new cucurbitane-type triterpenoids, named hemslepensides J-P (**1**–**7**), together with two known aglycones (**8**–**9**) (figure 1). In this paper, we elucidated the isolation and structure of seven new saponins. Moreover, based on our research results and previous published data [4–8], the relationships between the nuclear magnetic resonance (NMR) characteristics and the common substituents of cucurbitane tetracyclic triterpenes were discussed.

Compound **8** was first isolated in our laboratory and its cytotoxic activity has been screened in H460, SW620 and DU145 cell lines [6]. As the main component of *H. pengxianensis* var. *jinfushanensis*, compound **9** has been shown to inhibit human lung adenocarcinoma cell line A549 growth both *in vitro* and *in vivo* [9,10]. Although compounds **8** and **9** exhibit potent cytotoxicity, the underlying mechanism in colon cancer cells remains to be elucidated. In previous experiments, we screened the cytotoxicity of compounds **8** and **9**. The results revealed that these two compounds were more effective than cisplatin against colon cancer HT29 cells. So, the anti-colon cancer mechanism of compounds **8** and **9** was studied targeting the HT29 cell line.

## 2. Experimental procedure

### 2.1. Material and methods

The NMR spectra were acquired using a Bruker 600 Ultrashield NMR spectrometer (Bruker Biospin, Rheinstetten, Germany). The infrared (IR) spectra were measured using a Thermo Nicolet NEXUS 670 FTIR spectrometer (GMI, Ramsey, USA). The optical rotations were run on a JASCO P-2000 polarimeter (Jasco, Tokyo, Japan). High-resolution electrospray ionization mass spectrometry (HRESIMS) was measured on Thermo Scientific Accela PDA and LTQ-Orbitrap XL mass spectrometers (Thermo Fisher Scientific, Inc., Waltham, MA, USA). Preparative HPLC was run on an LC-6AD Shimadzu liquid chromatograph equipped with an SPD-20A UV/Vis detector (Shimadzu Corporation, Kyoto, Japan) and an ODS column (YMC-Pack ODS-A column, 250 × 20 mm, 5 µm, 12 nm, YMC Co., Ltd, Tokyo, Japan). The data of cell cycle and apoptosis were acquired using BD FACSCalibur flow cytometer (Becton, Dickinson and Company, NJ, USA). The optical density (OD) value was measured using a Victor 1420 multifunctional-counter instrument (Perkin Elmer, Waltham, MA, USA). Morphological changes were analysed under an FV3000 confocal laser scanning microscope (Olympus, Tokyo, Japan). Column chromatography (CC) was performed using an ODS C18 column (50 µm, YMC Co., Ltd, Tokyo, Japan) and silica gel (300–400 mesh, Qingdao Marine Chemistry Ltd, Qingdao, China). RPMI-1640, newborn calf serum and phosphate-buffered saline (PBS) were purchased from Hyclone (Logan, UT, USA). The trypsin/EDTA and Hoechst 33342 were purchased from Solarbio (Beijing Solarbio Science and Technology, Beijing, China). Thiazolyl blue tetrazolium bromide (MTT), d-glucose and l-glucose were purchased from Sigma-Aldrich (St Louis, USA). Propidium iodide (PI) cell cycle kit, Annexin V/PI apoptosis kit and Actin-Tracker Green kit were purchased from Beyotime Biotechnology (Shanghai, China).

### 2.2. Plant material

The rhizomes of *H. pengxianensis* were collected in September 2012 in Chongqing, China, and were identified by Dr Sirong Yi from the Chongqing Institute of Pharmaceutical Plants. Its voucher specimen (HA201209) was deposited in the Institute of Materia Medica of the Shandong Academy of Medical Science in China.

### 2.3. Extraction and isolation

The air-dried rhizomes (8.95 kg) of *H. pengxianensis* were decocted with 95% ethanol under refluxing each for 2 h three times. The total extraction was suspended in H_2_O and partitioned with EtOAc five times. The EtOAc fraction (650 g) was treated with silica gel CC (200–300 mesh, 10 × 120 cm, 2 kg) using a successive CH_2_Cl_2_–MeOH eluent (100 : 0→0 : 100, v/v) to give 10 fractions (A–J). Further isolation of Fr.H (35 g) was achieved via silica gel CC (CH_2_Cl_2_–MeOH, 99 : 1→0 : 100), repeated ODS gel CC (MeOH–H_2_O, 60 : 40 → 100 : 0) and prep-HPLC (MeOH–H_2_O, 80 : 20, 6.0 ml min^−1^, 210 nm) to give compound **1** (20 mg). Fr.I (71 g) was applied to silica gel CC (CH_2_Cl_2_–MeOH, 98 : 2 → 0 : 100) to yield subfractions (I1–I10). Fr.I5 (3.2 g), which was further purified by repeated ODS gel CC (MeOH–H_2_O, 50 : 50 → 100 : 0) followed by prep-HPLC (MeOH–H_2_O, 80 : 20, 73 : 27, respectively) to yield compounds **3** (15 mg), **4** (8 mg) and **2** (7.5 mg). The same methods with different proportions of eluent (prep-HPLC, MeOH–H_2_O, 68:32) were applied to Fr.I8 (6.5 g) to obtain compounds **5** (18 mg) and **6** (8 mg). Fr.I10 (2.7 g) was subjected to repeated ODS gel CC (MeOH–H_2_O, 40 : 60 → 100 : 0) followed by prep-HPLC (MeOH–H_2_O, 63 : 37) to give compound **7** (15 mg). Fr.B (236 g) was separated by silica gel CC using CH_2_Cl_2_–MeOH gradient mixtures (100 : 0 → 70 : 30) to afford six fractions (B1–B6). Fr.B3 (21.8 g) was repeated purified via ODS gel CC (MeOH–H_2_O, 45 : 55 → 70 : 30) to give compound **8** (4.1 g). Fr.B4 (155.2 g) was repeated crystallized in EtOAc to obtain compound **9** (121.3 g).

#### 2.3.1. Hemslepenside J (**1**)

C_42_H_68_O_13_, white amorphous powder; [*α*]^20^_D_ +96.95 (*c* 0.1, MeOH : H_2_O = 1:1); IR (KBr) *ν*_max_ 3378, 2937, 2874, 1697, 1462, 1374 and 1070 cm^−1^; ^1^H NMR and ^13^C NMR spectral data, see tables 1 and 2; HRESIMS *m/z* 798.5005 [M+NH_4_]^+^ (calcd. for C_42_H_68_O_13_NH_4_, 798.5004).

**Table 1.** The ^1^H NMR spectroscopic data of compounds **1**–**7** (*δ* in ppm, *J* in Hz).

no.	**1**^a^	**2**^b^	**3**^b^	**4**^b^	**5**^b^	**6**^b^	**7**^b^
1	1.06–1.09 (1H, m)	1.51–1.55 (1H, m)	1.53–1.57 (1H, m)	1.51–1.55 (1H, m)	1.31–1.35 (1H, m)	1.24–1.27 (1H, m)	1.22–1.25 (1H, m)
	1.42–1.49 (1H, m)	1.62–1.67 (1H, m)	2.19–2.25 (1H, m)	1.61–1.68 (1H, m)	2.17–2.25 (1H, m)	1.51–1.54 (1H, m)	1.52–1.57 (1H, m)
2	1.42–1.49 (1H, m)	1.78–1.84 (1H, m)	1.42–1.45 (1H, m)	1.81–1.84 (1H, m)	1.93–1.94 (1H, m)	1.32–1.37 (1H, m)	1.57–1.63 (1H, m)
	1.70–1.75 (1H, m)	2.05–2.09 (1H, m)	1.50–1.53 (1H, m)	2.05–2.10 (1H, m)	2.21–2.25 (1H, m)	1.98–2.02 (1H, m)	1.79–1.84 (1H, m)
3	3.28 (1H, br. s)	3.42 (1H, br. s)	3.41 (1H, br. s)	3.42 (1H, br. s)	3.42 (1H, br. s)	3.46 (1H, br. s)	3.43 (1H, br. s)
6	5.45–5.48 (1H, m)	5.54 (1H, dd, 5.8, 2.1)	5.49 (1H, dt, 6.5, 1.7)	5.54 (1H, dd, 6.2, 1.8)	5.49 (1H, d, 6.1)	5.61 (1H, dt, 4.3, 2.0)	5.63 (1H, d, 5.6)
7	1.84–1.86 (1H, m)	1.84–1.88 (1H, m)	1.82–1.84 (1H, m)	1.84–1.88 (1H, m)	1.81–1.83 (1H, m)	1.93–1.96 (1H, m)	1.91–1.95 (1H, m)
	2.24–2.31 (1H, m)	2.31–2.38 (1H, m)	2.39–2.45 (1H, m)	2.31–2.38 (1H, m)	2.35–2.42 (1H, m)	2.33–2.37 (1H, m)	2.37–2.40 (1H, m)
8	1.82–1.84 (1H, m)	1.93–1.94 (1H, m)	1.67 (1H, d, 7.5)	1.93–1.95 (1H, m)	1.67 (1H, d, 7.5)	1.93–1.96 (1H, m)	1.95–1.99 (1H, m)
10	2.33–2.39 (1H, m)	2.02–2.05 (1H, m)	2.49 (1H, dd, 12.2, 3.4)	2.02–2.05 (1H, m)	2.48 (1H, d, 12.3)	1.44–1.48 (1H, m)	2.40–2.44 (1H, m)
11		3.88 (1H, dd, 4.2, 2.0)	3.85 (1H, dd, 11.5, 5.5)	3.88 (1H, dd, 4.4, 2.1)	3.84 (1H, dd, 11.4, 5.1)		
12	2.21 (1H, d, 14.2)	1.97–2.02 (2H, m)	1.77–1.82 (2H, m)	1.97–2.02 (2H, m)	1.83–1.89 (2H, m)	2.38 (1H, d, 14.2)	2.47 (1H, d, 14.3)
	3.09 (1H, d, 14.2)					3.07 (1H, d, 14.2)	3.09 (1H, d, 14.3)
15	1.19–1.24 (1H, m)	1.21–1.26 (2H, m)	1.16–1.26 (2H, m)	1.21–1.26 (2H, m)	1.12–1.14 (1H, m)	1.28–1.32 (1H, m)	1.33–1.42 (2H, m)
	1.31–1.34 (1H, m)				1.20–1.23 (1H, m)	1.40–1.42 (1H, m)	
16	1.28–1.31 (1H, m)	1.28–1.30 (1H, m)	1.27–1.33 (1H, m)	1.26–1.31 (1H, m)	1.27–1.30 (1H, m)	1.38–1.40 (1H, m)	1.84–1.91 (2H, m)
	1.92–1.99 (1H, m)	1.88–1.91 (1H, m)	1.96–1.99 (1H, m)	1.88–1.91 (1H, m)	1.94–1.96 (1H, m)	1.74–1.77 (1H, m)	
17	1.65–1.70 (1H, m)	1.49–1.51 (1H, m)	1.57–1.63 (1H, m)	1.48–1.50 (1H, m)	1.61 (1H, q, 9.0)	1.77–1.79 (1H, m)	2.11 (1H, t, 9.5)
18	0.65 (3H, s)	1.02 (3H, s)	0.91 (3H, s)	1.02 (3H, s)	0.91 (3H, s)	0.75 (3H, s)	0.91 (3H, s)
19	0.96 (3H, s)	0.97 (3H, s)	1.15 (3H, s)	0.97 (3H, s)	1.11 (3H, s)	1.06 (3H, s)	1.10 (3H, s)
20	1.35–1.41 (1H, m)	1.46–1.48 (1H, m)	1.45–1.47 (1H, m)	1.45–1.47 (1H, m)	1.49–1.51 (1H, m)	2.40–2.45 (1H, m)	
21	0.87 (3H, d, 6.4)	0.98 (3H, d, 6.7)	0.97 (3H, d, 6.4)	0.98 (3H, d, 4.7)	0.98 (3H, d, 6.3)	0.94 (3H, d, 6.5)	1.26 (3H, s)
22	1.35–1.41 (1H, m)	1.04–1.14 (2H, m)	1.00–1.04 (1H, m)	1.03–1.13 (2H, m)	1.14–1.18 (1H, m)	1.10–1.14 (1H, m)	1.42–1.52 (2H, m)
	1.05–1.06 (1H, m)		1.06–1.09 (1H, m)		1.51–1.54 (1H, m)	1.48–1.51 (1H, m)	
23	1.86–1.89 (1H, m)	1.95–1.97 (1H, m)	1.99–2.05 (1H, m)	1.95–1.97 (1H, m)	1.76–1.81 (1H, m)	2.02–2.09 (1H, m)	2.06 (2H, q, 7.9)
	2.00–2.07 (1H, m)	2.09–2.15 (1H, m)	2.11–2.19 (1H, m)	2.12–2.19 (1H, m)	2.02–2.10 (1H, m)	2.17–2.24 (1H, m)	
24	5.40 (1H, t, 6.8)	5.47 (1H, t, 7.1)	5.37 (1H, t, 7.0)	5.38 (1H, t, 7.0)	5.63 (1H, t, 7.4)	5.55 (1H, t, 7.3)	5.49 (1H, t, 6.7)
26	3.89 (1H, d, 11.7)	4.04 (1H, d, 11.5)	1.79 (3H, s)	1.77 (3H, s)	4.16 (1H, d, 11.7)	4.08 (2H, s)	4.01 (1H, d, 11.5)
	4.11 (1H, d, 11.7)	4.20 (1H, d, 11.5)			4.38 (1H, d, 11.7)		4.20 (1H, d, 11.5)
27	1.60 (3H, s)	1.68 (3H, s)	4.27 (1H, d, 11.6)	4.19 (1H, d, 11.5)	4.17 (1H, d, 12.3)	4.16 (2H, s)	1.68 (3H, s)
			4.31 (1H, d, 11.6)	4.33 (1H, d, 11.5)	4.20 (1H, d, 12.3)		
28	0.95 (3H, s)	1.01 (3H, s)	1.10 (3H, s)	1.01 (3H, s)	1.06 (3H, s)	1.06 (3H, s)	1.04 (3H, s)
29	1.04 (3H, s)	1.20 (3H, s)	1.05 (3H, s)	1.20 (3H, s)	1.19 (3H, s)	1.23 (3H, s)	1.14 (3H, s)
30	1.00 (3H, s)	0.84 (3H, s)	0.86 (3H, s)	0.84 (3H, s)	0.87 (3H, s)	1.09 (3H,s)	1.11 (3H, s)

Glc′	26-Glc (inner)	3-Glc	27-Glc (inner)	3-Glc	3-Glc	3-Glc (inner)	26-Glc (inner)
1′	4.26 (1H, d, 7.8)	4.28 (1H, d, 7.8)	4.36 (1H, d, 7.7)	4.28 (1H, d, 7.8)	4.27 (1H, d, 7.8)	4.28 (1H, d, 7.8)	4.24 (1H, d, 7.9)
2′	3.24–3.27 (1H, m)	3.31–3.33 (1H, m)	3.45–3.49 (1H, m)	3.31–3.32 (1H, m)	3.19–3.20 (1H, m)	3.35–3.37 (1H, m)	3.18–3.21 (1H, m)
3′	3.14–3.18 (1H, m)	3.33–3.35 (1H, m)	3.52–3.56 (1H, m)	3.33–3.34 (1H, m)	3.31–3.33 (1H, m)	3.19–3.21 (1H, m)	3.38–3.42 (1H, m)
4′	3.07–3.11 (1H, m)	3.25–3.28 (1H, m)	3.35–3.37 (1H, m)	3.26–3.29 (1H, m)	3.25–3.30 (1H, m)	3.26–3.28 (1H, m)	3.34–3.35 (1H, m)
5′	3.11–3.14 (1H, m)	3.20–3.23 (1H, m)	3.21–3.23 (1H, m)	3.20–3.24 (1H, m)	3.25–3.30 (1H, m)	3.29–3.30 (1H, m)	3.21–3.27 (1H, m)
6′	3.63–3.68 (1H, m)	3.65–3.68 (1H, m)	3.67–3.70 (1H, m)	3.67–3.71 (1H,m)	3.65–3.68 (1H, m)	3.78 (1H, dd, 12.0, 6.0)	3.78 (1H, dd, 12.1, 5.0)
	3.45–3.50 (1H, m)	3.84–3.87 (1H, m)	3.83–3.85 (1H, m)	3.84–3.87 (1H, m)	3.83–3.88 (1H, m)	4.07 (1H, dd, 12.0, 1.9)	4.13 (1H, dd, 12.1, 1.9)

Glc″	26-Glc (terminal)	26-Glc	27-Glc (terminal)	27-Glc	26-Glc	3-Glc (terminal)	26-Glc (terminal)
1″	4.40 (1H, d, 7.8)	4.24 (1H, d, 7.9)	4.62 (1H, d, 7.8)	4.21 (1H, d, 7.8)	4.28 (1H, d, 7.8)	4.41 (1H, d, 7.8)	4.39 (1H, d, 7.8)
2″	2.97–3.01 (1H, m)	3.16–3.18 (1H, m)	3.24–3.26 (1H, m)	3.16–3.20 (1H,m)	3.17–3.19 (1H, m)	3.17–3.19 (1H, m)	3.32–3.34 (1H, m)
3″	3.02–3.05 (1H, m)	3.28–3.30 (1H, m)	3.23–3.24 (1H, m)	3.31–3.34 (1H,m)	3.31–3.33 (1H, m)	3.39–3.43 (1H, m)	3.35–3.38 (1H, m)
4″	3.07–3.11 (1H, m)	3.18–3.20 (1H, m)	3.27–3.30 (1H, m)	3.20–3.24 (1H,m)	3.21–3.25 (1H, m)	3.32–3.34 (1H, m)	3.28 (1H, d, 8.2)
5″	3.35–3.37 (1H, m)	3.20–3.23 (1H, m)	3.31–3.34 (1H, m)	3.16–3.20 (1H,m)	3.20–3.21 (1H, m)	3.28–3.29 (1H, m)	3.21–3.27 (1H, m)
6″	3.58–3.63 (1H, m)	3.63–3.65 (1H, m)	3.63–3.67 (1H, m)	3.63–3.67 (1H,m)	3.63–3.65 (1H, m)	3.67 (1H, dd, 11.9, 5.2)	3.66 (1H, dd, 11.9, 5.5)
	3.40–3.45 (1H, m)	3.81–3.84 (1H, m)	3.80–3.83 (1H, m)	3.81–3.84 (1H, m)	3.79–3.83 (1H, m)	3.87 (1H, dd, 11.9, 2.1)	3.86 (1H, dd, 11.9, 2.1)

^a^^1^H NMR data measured in DMSO-*d*_6_ at 600 MHz.

^b^^1^H NMR data measured in MeOH-*d*_4_ at 600 MHz.

**Table 2.** The ^13^C NMR (150 MHz) spectroscopic data for compounds **1**–**7**.

no.	**1**^a^	**2**^b^	**3**^b^	**4**^b^	**5**^b^	**6**^b^	**7**^b^
	*δ*_c_, type	*δ*_c_, type	*δ*_c_, type	*δ*_c_, type	*δ*_c_, type	*δ*_c_, type	*δ*_c_, type
1	20.2, CH_2_	24.4, CH_2_	26.6, CH_2_	24.4, CH_2_	27.4, CH_2_	22.7, CH_2_	21.8, CH_2_
2	28.5, CH_2_	29.7, CH_2_	30.6, CH_2_	29.7, CH_2_	29.7, CH_2_	29.1, CH_2_	29.9, CH_2_
3	74.2, CH	88.6, CH	78.0, CH	88.6, CH	88.7, CH	87.8, CH	77.1, CH
4	41.0, C	42.6, C	42.8, C	42.6, C	43.0, C	42.7, C	42.4, C
5	140.9, C	143.8, C	144.1, C	143.8, C	145.1, C	142.2, C	141.5, C
6	117.5, CH	120.5, CH	120.7, CH	120.5, CH	119.8, CH	119.8, CH	120.6, CH
7	23.5, CH_2_	25.4, CH_2_	25.2, CH_2_	25.4, CH_2_	25.2, CH_2_	25.0, CH_2_	24.9, CH_2_
8	43.2, CH	43.0, CH	44.9, CH	43.0, CH	44.8, CH	45.5, CH	44.8, CH
9	48.1, C	41.4, C	41.0, C	41.4, C	41.2, C	50.5, C	50.3, C
10	34.5, CH	41.0, CH	37.2, CH	41.0, CH	37.4, CH	37.0, CH	36.8, CH
11	213.5, C	74.0, CH	79.4, CH	74.0, CH	79.5, CH	217.9, C	217.9, C
12	48.3, CH_2_	39.5, CH_2_	41.2, CH_2_	39.5, CH_2_	41.0, CH_2_	49.7, CH_2_	50.1, CH_2_
13	48.4, C	46.4, C	48.4, C	46.4, C	48.4, C	50.2, C	51.3, C
14	49.0, C	50.8, C	50.7, C	50.8, C	50.7, C	50.9, C	50.3, C
15	33.9, CH_2_	36.1, CH_2_	35.5, CH_2_	36.1, CH_2_	35.5, CH_2_	35.6, CH_2_	35.1, CH_2_
16	27.3, CH_2_	29.0, CH_2_	29.3, CH_2_	29.0, CH_2_	29.2, CH_2_	29.0, CH_2_	22.9, CH_2_
17	48.7, CH	52.3, CH	51.8, CH	52.3, CH	51.7, CH	50.7, CH	52.2, CH
18	16.6, CH_3_	18.1, CH_3_	17.3, CH_3_	18.0, CH_3_	17.3, CH_3_	17.4, CH_3_	19.4, CH_3_
19	19.6, CH_3_	22.6, CH_3_	26.4, CH_3_	22.6, CH_3_	26.3, CH_3_	20.4, CH_3_	20.4, CH_3_
20	35.1, CH	37.3, CH	37.3, CH	37.3, CH	37.2, CH	37.0, CH	75.8, C
21	18.2, CH_3_	19.2, CH_3_	19.4, CH_3_	19.3, CH_3_	19.2, CH_3_	19.0, CH_3_	26.1, CH_3_
22	35.5, CH_2_	37.3, CH_2_	37.9, CH_2_	38.0, CH_2_	37.5, CH_2_	37.4, CH_2_	44.9, CH_2_
23	23.7, CH_2_	25.7, CH_2_	25.8, CH_2_	25.7, CH_2_	25.4, CH_2_	25.2, CH_2_	23.8, CH_2_
24	127.0, CH	130.9, CH	132.0, CH	132.1, CH	134.6, CH	130.9, CH	130.2, CH
25	131.5, C	132.7, C	132.5, C	132.4, C	136.2, C	139.2, C	132.9, C
26	73.5, CH_2_	76.1, CH_2_	22.1, CH_3_	22.0, CH_3_	73.2, CH_2_	65.7, CH_2_	76.2, CH_2_
27	13.7, CH_3_	14.3, CH_3_	68.4, CH_2_	68.0, CH_2_	58.5, CH_2_	58.4, CH_2_	14.3, CH_3_
28	27.5, CH_3_	28.6, CH_3_	27.6, CH_3_	28.7, CH_3_	28.0, CH_3_	28.8, CH_3_	28.5, CH_3_
29	25.7, CH_3_	26.1, CH_3_	26.6, CH_3_	26.1, CH_3_	26.5, CH_3_	26.0, CH_3_	26.2, CH_3_
30	17.7, CH_3_	18.7, CH_3_	20.0, CH_3_	18.7, CH_3_	19.9, CH_3_	19.0, CH_3_	19.2, CH_3_

Glc′	26-Glc (inner)	3-Glc	27-Glc (inner)	3-Glc	3-Glc	3-Glc (inner)	26-Glc (inner)
1′	100.2, CH	106.8, CH	101.4, CH	106.8, CH	106.7, CH	106.5, CH	102.9, CH
2′	82.0, CH	75.7, CH	82.1, CH	75.7, CH	75.7, CH	75.7, CH	75.2, CH
3′	76.1, CH	78.4, CH	78.4, CH	78.4, CH	78.4, CH	78.1, CH	77.2, CH
4′	69.8, CH	71.8, CH	71.7, CH	71.8, CH	71.8, CH	71.8, CH	71.6, CH
5′	76.7, CH	78.0, CH	77.9, CH	78.0, CH	78.1, CH	78.3, CH	78.2, CH
6′	60.8, CH_2_	63.0, CH_2_	62.8, CH_2_	63.0, CH_2_	63.0, CH_2_	70.0, CH_2_	69.8, CH_2_

Glc″	26-Glc (terminal)	26-Glc	27-Glc (terminal)	27-Glc	26-Glc	3-Glc (terminal)	26-Glc (terminal)
1″	104.1, CH	102.6, CH	104.9, CH	102.6, CH	102.9, CH	105.0, CH	104.9, CH
2″	75.0, CH	75.2, CH	76.0, CH	75.2, CH	75.2, CH	75.3, CH	75.1, CH
3″	76.9, CH	78.3, CH	78.3, CH	78.3, CH	78.3, CH	77.3, CH	78.1, CH
4″	69.7, CH	71.8, CH	71.5, CH	71.7, CH	71.8, CH	71.7, CH	71.7, CH
5″	76.2, CH	77.8, CH	77.7, CH	77.8, CH	77.8, CH	78.1, CH	78.2, CH
6″	60.9, CH_2_	62.9, CH_2_	62.9, CH_2_	62.8, CH_2_	62.9, CH_2_	62.9, CH_2_	62.9, CH_2_

^a^^13^C NMR data measured in DMSO-*d*_6_ at 150 MHz.

^b^^13^C NMR data measured in MeOH-*d*_4_ at 150 MHz.

#### 2.3.2. Hemslepenside K (**2**)

C_42_H_70_O_13_, white amorphous powder; [*α*]^20^_D_ −21.77 (*c* 0.1, MeOH); IR (KBr) *ν*_max_ 3417, 2926, 2866, 1075 and 1019 cm^−1^; ^1^H NMR and ^13^C NMR spectral data, see tables 1 and 2; HRESIMS *m/z* 805.4705 [M+Na]^+^ (calcd. for C_42_H_70_O_13_Na, 805.4714).

#### 2.3.3. Hemslepenside L (**3**)

C_42_H_70_O_13_, white amorphous powder; [*α*]^20^_D_ +32.33 (*c* 0.1, MeOH); IR (KBr) *ν*_max_ 3417, 2932, 2871, 1076 and 1025 cm^−1^; ^1^H NMR and ^13^C NMR spectral data, see tables 1 and 2; HRESIMS *m/z* 805.4714 [M+Na]^+^ (calcd. for C_42_H_70_O_13_Na, 805.4714).

#### 2.3.4. Hemslepenside M (**4**)

C_42_H_70_O_13_, white amorphous powder; [*α*]^20^_D_ +19.89 (*c* 0.1, MeOH); IR (KBr) *ν*_max_ 3385, 2935, 2866, 1074 and 1020 cm^−1^; ^1^H NMR and ^13^C NMR spectral data, see tables 1 and 2; HRESIMS *m/z* 805.4710 [M+Na]^+^ (calcd. for C_42_H_70_O_13_Na, 805.4714).

#### 2.3.5. Hemslepenside N (**5**)

C_42_H_70_O_14_, white amorphous powder; [*α*]^20^_D_ +17.35 (*c* 0.1, MeOH); IR (KBr) *ν*_max_ 3384, 2941, 2866, 1076 and 1029 cm^−1^; ^1^H NMR and ^13^C NMR spectral data, see tables 1 and 2; HRESIMS *m/z* 799.4846 [M+H]^+^ (calcd. for C_42_H_71_O_14_, 799.4844).

#### 2.3.6. Hemslepenside O (**6**)

C_42_H_68_O_14_, white amorphous powder; [*α*]^20^_D_ +93.49 (*c* 0.1, MeOH); IR (KBr) *ν*_max_ 3386, 2930, 2866, 1691, 1075 and 1033 cm^−1^; ^1^H NMR and ^13^C NMR spectral data, see tables 1 and 2; HRESIMS *m/z* 814.4963 [M+NH_4_]^+^ (calcd. for C_42_H_68_O_14_NH_4_, 814.4953).

#### 2.3.7. Hemslepenside P (**7**)

C_42_H_68_O_14_, white amorphous powder; [*α*]^20^_D_ +44.43 (*c* 0.1, MeOH); IR (KBr) *ν*_max_ 3419, 2929, 2877, 1686, 1076 and 1032 cm^−1^; ^1^H NMR and ^13^C NMR spectral data, see tables 1 and 2; HRESIMS *m/z* 814.4964 [M+NH_4_]^+^ (calcd. for C_42_H_68_O_14_NH_4_, 814.4953).

### 2.4. Enzymatic hydrolysis of **1**–**7** and determination of the absolute configuration of the monosaccharides

To each solution of compounds **1**–**7** (4 mg) in H_2_O (4 ml) was added cellase (each 20 mg) and stirred at 40°C for 120 h. The reaction mixture was partitioned between EtOAc and H_2_O and the EtOAc layer was separated. The aqueous layer was evaporated under reduced press to afford solid saccharide mixture. The authentic samples of d-(+)-glucose and l-(−)-glucose and the saccharide mixture were dissolved in 1 ml of H_2_O and mixed with 1 ml of EtOH to which (*S*)-(−)-*α*-methylbenzylamine (7 µl) and NaBH_3_CN (6.75 mg) were added, respectively. The mixture was stirred at 40°C for 4 h followed by the addition of glacial acetic acid (0.2 ml) and evaporated under reduced pressure to afford solid mixture. To the solid mixture was added acetic anhydride (0.3 ml) in pyridine (0.3 ml) for acetylation for 24 h at room temperature. H_2_O (1 ml) was added to the reaction mixture and evaporated under reduced pressure to remove residual pyridine (repeated three times). The residue was suspended in H_2_O (0.5 ml) and subjected to a Cleanert C18-N column (Agela) eluented with H_2_O, 20% CH_3_CN and 50% CH_3_CN (15, 15 and 5 ml) successively in order to obtain 50% CH_3_CN fraction. The 50% CH_3_CN fraction was monitored at 210 nm by HPLC under the following conditions: Agilent SB-C18 column (4.6 × 250 mm, 5 µm); 40% CH_3_CN/H_2_O; 0.8 ml min^−1^; 30°C. The composition sugar of compounds **1**–**7** was determined to be d-(+)-glucose by comparing the retention time *t*_R_ (min) of their 1-[(*S*)-*N*-acetyl-*a*-methylbenzylamino]-1-deoxyglucitol acetate derivatives with those of authentics: 20.8 min (derivative of d-glucose) and 19.4 min (derivative of l-glucose).

### 2.5. Cell culture and cytotoxicity assay

The HT29 cell lines were purchased from the Institute of Materia Medica of the Chinese Academy of Medical Sciences. The cells were cultured in RPMI-1640 containing 10% newborn calf serum at 37°C in a humidified 5% CO_2_ air. The cells were detached using a solution of 0.25% trypsin/EDTA. Approximately 6 × 10^3^ cells per well were plated in 96-well plates. After incubating for 24 h, cisplatin, DMSO (2‰) and isolated compounds (100, 10, 1, 0.1, 0.01 and 0 µM) were added for another 48 h, respectively. Then, the cells per well were treated with 10 µl of MTT solution (5 mg ml^−1^ in PBS) and reincubated for 4 h at 37°C. The medium was removed and 150 µl well^−1^ DMSO was replaced. The plates were oscillated gently for 5–10 min to dissolve fully in a 96-well plate oscillator. The OD value of each well was measured at 570 nm using the Victor 1420 instrument, and the half-maximal inhibitory concentration (IC_50_) values were calculated using the SPSS statistical software. Cisplatin served as a positive control. At least, three independent experiments were performed.

### 2.6. Flow cytometric analysis for cell cycle distribution

The 3 × 10^5^ well^−1^ HT29 cells were seeded into six-well plates in 2 ml of medium and incubated at 37°C overnight. Then, the HT29 cells were treated with DMSO (2‰), compounds **8** and **9** (0, 0.1 or 1 µM, respectively) for 24 h. Afterwards, the cells were collected by trypsinization and fixed in 70% ice-cold ethanol at 4°C for another 24 h. The cells were then washed twice with PBS and stained with fluorochrome solution including PI and RNAse in the dark for 30 min at 37°C. The DNA content was analysed by flow cytometer. At least three independent experiments were performed.

### 2.7. Cell apoptosis detection

The HT29 cells were plated at a concentration of 3 × 10^5^ cells per well in 2 ml of medium in six-well plates overnight. Then, the cells were treated with either 0.1 or 1 µM compounds **8** and **9**, respectively, for 24 h. Following harvesting by trypsinization, the cells were washed once with PBS and then were resuspended in 195 µl of Annexin V-FITC binding buffer. Afterwards, 5 µl of Annexin V-FITC and 10 µl of PI were added in turn followed by incubating in the dark for 15 min at room temperature (20–25°C). The cells were analysed using flow cytometer. At least three independent experiments were performed.

### 2.8. Fluorescence microscopy

Approximately 5 × 10^4^ HT29 cells per well were grown on sterilized glass coverslips. After the treatment with either 0.1 or 1 µM compounds **8** and **9**, respectively, for 24 h, the cells were fixed with beyotime P0098 fixative (15 min) and then washed three times with beyotime P0106 clearing solution (5 min each time). The actin-tracker green staining solution (200 µl well^−1^) was added to the coverslips in the dark and incubated for 60 min at room temperature. The stained cells were rinsed three times with beyotime P0106 clearing solution (5 min each time) followed by staining using the Hoechst 33342 for 5 min and washed three times (3 min each time) in PBS. Morphological changes were analysed under an FV3000 confocal laser scanning microscope.

### 2.9. Statistical analysis

All statistical comparisons were made by Statistical Product and Service Solutions (SPSS, v. 20.0). The differences were described as statistically significant if *p* < 0.05.

## 3. Results and discussion

### 3.1. The structure elucidation of seven new saponins

**Figure 2.**
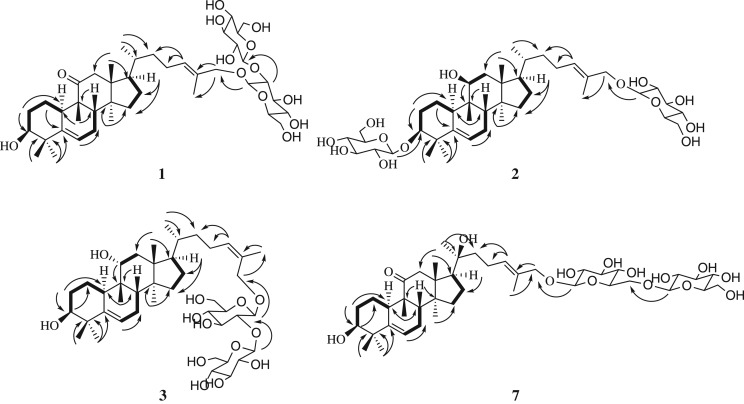
Key ^1^H-^1^H COSY (bold bonds) and HMBC (arrows) correlations for compounds **1**–**3** and **7**.

**Figure 3.**
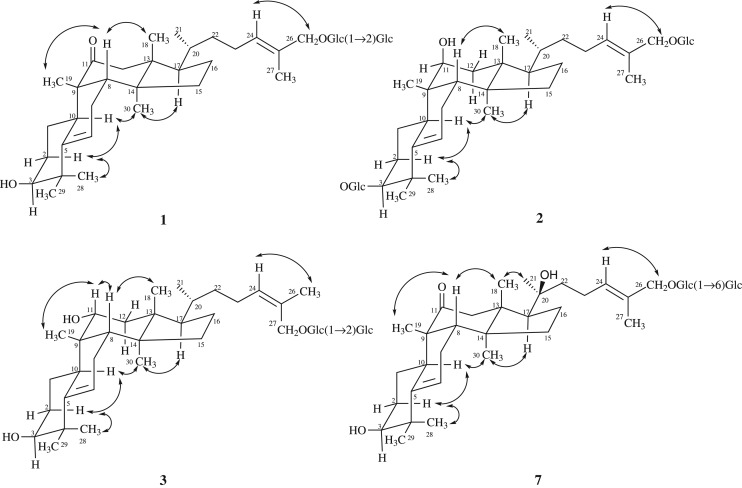
Key NOESY correlations of compounds **1**–**3** and **7**.

Hemslepenside J (**1**) was obtained as a white solid. The molecular formula was determined as C_42_H_68_O_13_ on the basis of the HRESIMS data analysis (*m/z* 798.5005 [M+NH_4_]^+^, calcd. for 798.5004). The IR absorption bands at 3378, 2937 and 1697 cm^−1^ indicated the presence of hydroxyl, methyl, methylene and carbonyl groups. Comparison of the NMR data of **1** (tables 1 and 2) with those of delavanoside A indicated that these two compounds have the same aglycone and they just differ in the attachment point of the sugar residue [11]. The sugar residue was attached to C-26 (*δ*_C_ 73.5) in **1**, which was confirmed by the HMBC correlations from *δ*_H_ 4.26 (1H, d, *J* = 7.8 Hz, H-1′) to *δ*_C_ 73.5 (C-26) (figure 2). The equatorial configuration of H-3 could be deduced from its couple constant *J*_3eq, 2ax_ = *J*_3eq, 2eq_ and an obvious broad singlet of oxygenated methine proton at *δ*_H_ 3.28 (H-3). The relative configuration of **1** was determined via NOESY data (figure 3), in which correlations of H-28/H-10, H-10/H-30 and H-30/H-17 indicated the α-orientation of H-10, H-17 and CH_3_-30. Meanwhile, the NOESY correlations of H-19/H-8 and H-8/H-18 indicated the β-orientation of H-8, CH_3_-18 and CH_3_-19. Enzymatic hydrolysis of **1** afforded d-glucose as the composition sugar via the HPLC analysis of its 1-[(*S*)-*N*-acetyl-*α*-methylbenzylamino]-1-deoxyglucitol acetate derivatives. The large coupling constant value 7.8 Hz (*J*_H-1′,H-2′_ and *J*_H-1″,H-2″_) of anomeric protons showed the presence of two *β*-glucopyranosyl moieties. The *trans* configuration of Δ^24^ was confirmed by the NOESY correlation of H-24/H-26 (figure 3). Thus, compound **1** was determined to be 3*β*,26-dihydroxycucurbita-5,24(*E*)-diene-11-one-26-*O*-*β*-d-glucopyranosyl(1 → 2)-*β*-d-glucopyranoside.

Hemslepenside K (**2**) was isolated as a white solid. The molecular formula was determined as C_42_H_70_O_13_ from the molecular ion peaks [M+Na]^+^ at *m/z* 805.4705 (calcd. for C_42_H_70_O_13_Na, 805.4714) in the positive-mode HRESIMS. The NMR data of **2** (tables 1 and 2) were almost identical with that of hemslepenside H except for C-11 which could be ascribed to the α-orientation hydroxyl of C-11 in hemslepenside H replaced by β-orientation hydroxyl of C-11 in **2** [8]. Above deduction was confirmed by the two small coupling constants 4.2 Hz (*J*_H-11,Ha-12_) and 2.0 Hz (*J*_H-11,Hb-12_) of *δ*_H_ 3.88 (1H, dd, H-11). In addition, the *trans* configuration of the Δ^24^ was determined via the NOESY correlations from *δ*_H_ 5.47 (1H, t, *J* = 7.1 Hz, H-24) to *δ*_H_ 4.04 (1H, d, *J* = 11.5 Hz, H_a_-26) and 4.20 (1H, d, *J* = 11.5 Hz, H_b_-26). Thus, the structure of **2** was determined to be 3-*O*-*β*-d-glucopyranosyl-3*β*,11*β*,26-trihydroxycucurbita-5,24(*E*)-diene-26-*O*-*β*-d-glucopyranoside.

Hemslepenside L (**3**) was isolated as a white solid. The molecular formula C_42_H_70_O_13_ was determined via its HRESIMS data (*m/z* 805.4714 [M+Na]^+^, calcd. for 805.4714), which suggested that **3** and hemslepenside H were a pair of isomers [8]. The ^13^C NMR data of **3** (table 2) and hemslepenside H were almost identical with the exception of the *β*-d-glucose link location. Two *β*-d-glucoses were linked to C-3 and C-26, respectively, in hemslepenside H, whereas two *β*-d-glucoses composed a disaccharide unit, then linked to C-27 in 3, which could be confirmed by HMBC correlations from *δ*_H_ 4.36 (1H, d, *J* = 7.7 Hz, H-1′) to *δ*_C_ 68.4 (C-27), from *δ*_H_ 4.62 (1H, d, *J* = 7.8 Hz, H-1″) to *δ*_C_ 82.1 (C-2′). The relative configuration of **3** was deduced from NOESY data (figure 3), especially the NOESY correlations from *δ*_H_ 5.37 (1H, t, *J* = 7.0 Hz, H-24) to *δ*_H_ 1.79 (3H, s, H-26) showed that the terminal double bond was the *cis* configuration. Thus, the structure of **3** was determined to be 3*β*,11*α*,27-trihydroxycucurbita-5,24(*Z*)-diene-27-*O*-*β*-d-glucopyranosyl(1 → 2)-*β*-d-glucopyranoside.

Hemslepenside M (**4**) was isolated as a white solid and an isomer of compound **2** according to the HRESIMS data (*m/z* 805.4710 [M+Na]^+^, calcd. for 805.4714). The ^1^H and ^13^C NMR spectra (tables 1 and 2) displayed one β-orientation hydroxyl at C-11 (*δ*_C_ 74.0), and two β-d-glucoses linked at C-3 (*δ*_C_ 88.6) and C-27 (*δ*_C_ 68.0), respectively. The above deduction was confirmed by the two small coupling constants 4.4 Hz (*J*_H-11,Ha-12_) and 2.1 Hz (*J*_H-11,Hb-12_) of *δ*_H_ 3.88 (H-11), and the HMBC correlations from H-1′ (*δ*_H_ 4.28, 1H, d, *J* = 7.8 Hz) to *δ*_C_ 88.6 (C-3) and from H-1″ (*δ*_H_ 4.21, 1H, d, *J* = 7.8 Hz) to *δ*_C_ 68.0 (C-27). The structure and relative configuration of **4** were almost identical to those of **2** by comparing the NMR and NOESY data (figure 3). The significant differences, the NOESY correlation between *δ*_H_ 5.38 (1H, t, *J* = 7.0 Hz, H-24) and 1.77 (3H, s, H-26) in 4, emerged at C-26, which further confirmed the *cis* double bond at C-24 in **4** instead of the *trans* one in **2**. Thus, the structure of **4** was determined to be 3-*O*-*β*-d-glucopyranosyl-3*β*,11*β*,27-trihydroxycucurbita-5,24(*Z*)-diene-27-*O*-*β*-d-glucopyranoside.

Hemslepenside N (**5**) was obtained as a white solid. It showed a positive ion peak at *m/z* 799.4846 [M+H]^+^ (calcd. for 799.4844), which determined the molecular formula of **5** as C_42_H_70_O_14_. The molecular weight of **5** was 16 mass units more than that of hemslepenside H [8], which implied an additional hydroxyl unit in **5**. The significant difference was a hydroxymethyl signal at *δ*_C_ 58.5 (C-27) in **5** but a methyl signal at *δ*_C_ 15.0 (C-27) in hemslepenside H, which was consistent with the results of molecular weight analysis. In addition, the 24(*E*)-ene was deduced from the NOESY correlation of H-24/H-26. Thus, the structure of **5** was determined to be 3-*O*-*β*-d-glucopyranosyl-3*β*,11*α*,26,27-tetrahydroxycucurbita-5,24(*E*)-diene-26-*O*-*β*-d-glucopyranoside.

Hemslepenside O (**6**) was isolated as a white amorphous powder and had a molecular formula of C_42_H_68_O_14_ according to positive ion HRESIMS (*m/z* 814.4963 [M+NH_4_]^+^, calcd. for 814.4953). The molecular weight of **6** was 162 mass units more than that of jinfushanoside B [4], which suggested an additional hexose unit existed in **6**. The additional *β*-d-glucose was connected to C-6′, which could be deduced from the HMBC correlations from *δ*_H_ 4.41 (1H, d, *J* = 7.8 Hz, H-1″) to *δ*_C_ 70.0 (C-6′). The same NOESY and HMBC correlations for the 26,27-dihydroxycucurbita-11-one skeleton as those in jinfushanoside B were found. Thus, the structure of **6** was assigned as 3*β*,26,27-trihydroxycucurbita-5,24-diene-11-one-3-*O*-*β*-*D*-glucopyranosyl(1 → 6)-*β*-*D*-glucopyranoside.

Hemslepenside P (**7**) was isolated as a white amorphous powder. The elemental formula for **7** was assigned as C_42_H_68_O_14_ from the ion peak [M+NH_4_]^+^ at *m/z* 814.4964 (calcd. for 814.4953) in the positive-mode HRESIMS. In the ^13^C NMR and DEPT spectra, the significant difference between **7** and carnosifloside I came from the great downfield of C-20 (from *δ*_C_ 35.9 in carnosifloside I to 75.8 in **7**), which indicated the attachment of a hydroxyl to C-20 in **7** [12]. The above deduction was confirmed by its 16 mass units more than that of carnosifloside I and supported by HMBC correlations from *δ*_H_ 1.26 (3H, s, H-21) to *δ*_C_ 75.8 (C-20), 44.9 (C-22) and 52.2 (C-17). The NOESY correlations from *δ*_H_ 0.91 (3H, s, H-18) to 1.26 (3H, s, H-21) indicated the *S*-configuration of C-20. Thus, the structure of **7** was established as 3*β*,20*S*,26-trihydroxycucurbita-5,24(*E*)-diene-11-one-26-*O*-*β*-d-glucopyranosyl(1→6)-*β*-d-glucopyranoside.

### 3.2. Cell experiments

#### 3.2.1. Growth inhibitory properties of compounds **8** and **9** in HT29 cells

Seven new compounds (**1**–**7**) and two known compounds (**8**, **9**) were evaluated for cytotoxicity in HT29 cell line. HT29 cells were treated with different concentrations (0, 0.01, 0.1, 1, 10 or 100 µM) of **1**–**9** for 48 h, or treated with DMSO (2‰) as solvent control. After 48 h of treatment, their IC_50_ values are shown in table 3. Compounds **8** and **9** displayed significant cytotoxicity; however, except compound **2**, the other six new compounds had no cytotoxicity. The results suggest that the cytotoxicity of cucurbitane tetracyclic triterpenes sapogenins is generally more potent than that of saponins based on previous reports of our group [6–8].

**Table 3.** The IC_50_ values of compounds **1**–**9** against HT29 cell line.

compound code	HT29 cell line
**1**	>100
**2**	17.71 ± 0.95
**3**	>100
**4**	>100
**5**	>100
**6**	>100
**7**	>100
**8**	0.69 ± 0.06
**9**	0.37 ± 0.025
cisplatin	4.87 ± 0.38

#### 3.2.2. The effects of compounds **8** and **9** on the G_2_/M phase of the cell cycle in HT29 cells

**Figure 4.**
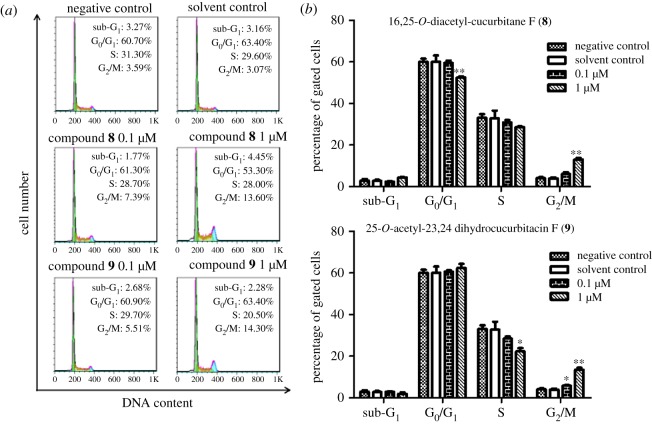
Effect of 16,25-*O*-diacetyl-cucurbitane F (**8**) and 25-*O*-acetyl-23,24-dihydrocucurbitacin F (**9**) on cell cycle distribution. (*a*) Representative DNA histograms of cell cycle analysis in HT29 cells. The cells were treated with either **8** (0, 0.1 and 1.0 µM), **9** (0.1 and 1.0 µM) or DMSO (2‰) for 24 h, stained with PI and analysed by flow cytometry, respectively. (*b*) Graphical representation of the cell cycle phase distribution. The results show the mean ± s.e.m. of three independent experiments. Significantly different (*p < 0.05, **p < 0.01) compared with negative control (0 µM-treated).

To investigate the underlying mechanism of the proliferation inhibitory effects of compounds **8** and **9** in HT29 cells, the cell cycle check points were examined by flow cytometry. HT29 cells were treated with 0.1 and 1 µM of compounds **8** and **9** for 24 h in the experimental group, while the control group was treated with the DMSO (2‰). As shown in figure 4, treatment with DMSO (2‰) had no effect on the distribution of cell cycle. Compared with the control group, the percentage of cells in the G_2_/M phase increased significantly with the increasing dose of **8** and **9**. After treatment with 1 µM compound **8** for 24 h, the percentage of cells in the G_2_/M phase increased from 4.1% to 13.1%. Compound **9** showed similar effects to compound **8**. These results suggested that induction of G_2_/M cell cycle arrest may contribute to the inhibitory effects of compounds **8** and **9** on cell proliferation.

#### 3.2.3. The effects of compounds **8** and **9** on apoptotic induction in HT29 cells

**Figure 5.**
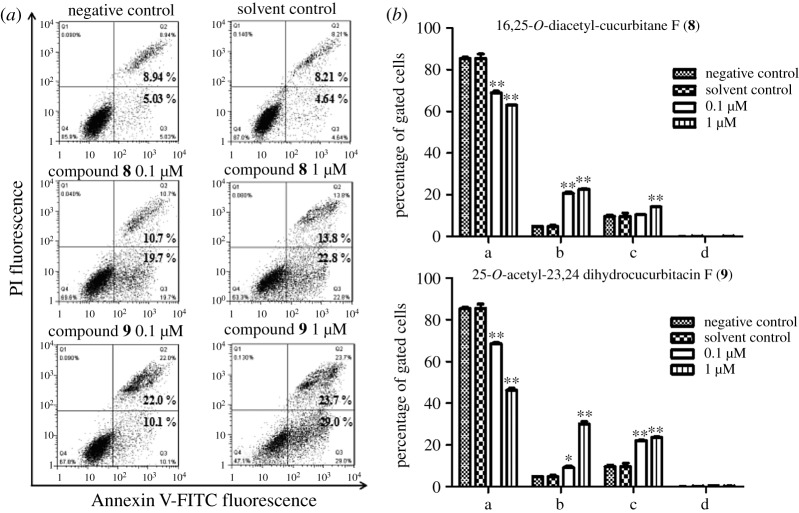
Effect of 16,25-*O*-diacetyl-cucurbitane F (**8**) and 25-*O*-acetyl-23,24-dihydrocucurbitacin F (**9**) on cell apoptosis. (*a*) Density plots showing the percentage distribution of compounds **8** and **9** treated cells. The HT29 cells were stained with Annexin V/PI and analysed by flow cytometry. Upper left quadrant: Annexin V(−)/PI(+) are labelled as damaged cells. Upper right quadrant: Annexin V(+)/PI(+) are labelled as late apoptotic/secondary necrotic cells. Lower right quadrant: Annexin V(+)/PI(−) are labelled as early apoptotic cells. Lower left quadrant: Annexin V(−)/PI(−) are labelled as live cells. (*b*) Graphical representation of the cell apoptosis phase distribution. The results show the mean ± s.e.m. of three independent experiments. Significantly different (*p < 0.05, **p < 0.01) compared with negative control (0 µM-treated). The abscissa ‘a, b, c and d’ represents in turn ‘live cells, early apoptotic cells, late apoptotic/secondary necrotic cells, and damaged cells'.

To determine if apoptosis is involved in the two compounds' induced inhibition of cell proliferation, we employed Annexin V/PI double staining to examine the effect of compounds **8** and **9** on apoptosis. The results indicated that treating with 0.1 and 1 µM compounds **8** or **9** for 24 h induced a significant increase in the percentage of apoptotic and necrotic cells when compared with the control group. As shown in figure 5, DMSO (2‰) did not induce cell apoptosis, while compounds **8** and **9** significantly increased the percentage of apoptotic and necrotic cells in a dose-dependent manner. These results suggested that induction of cell apoptosis may be involved in the inhibitory effects of compounds **8** and **9** on cell proliferation in HT29 cells.

#### 3.2.4. Induction of cell morphological changes by compounds **8** and **9** in HT29 cells

**Figure 6.**
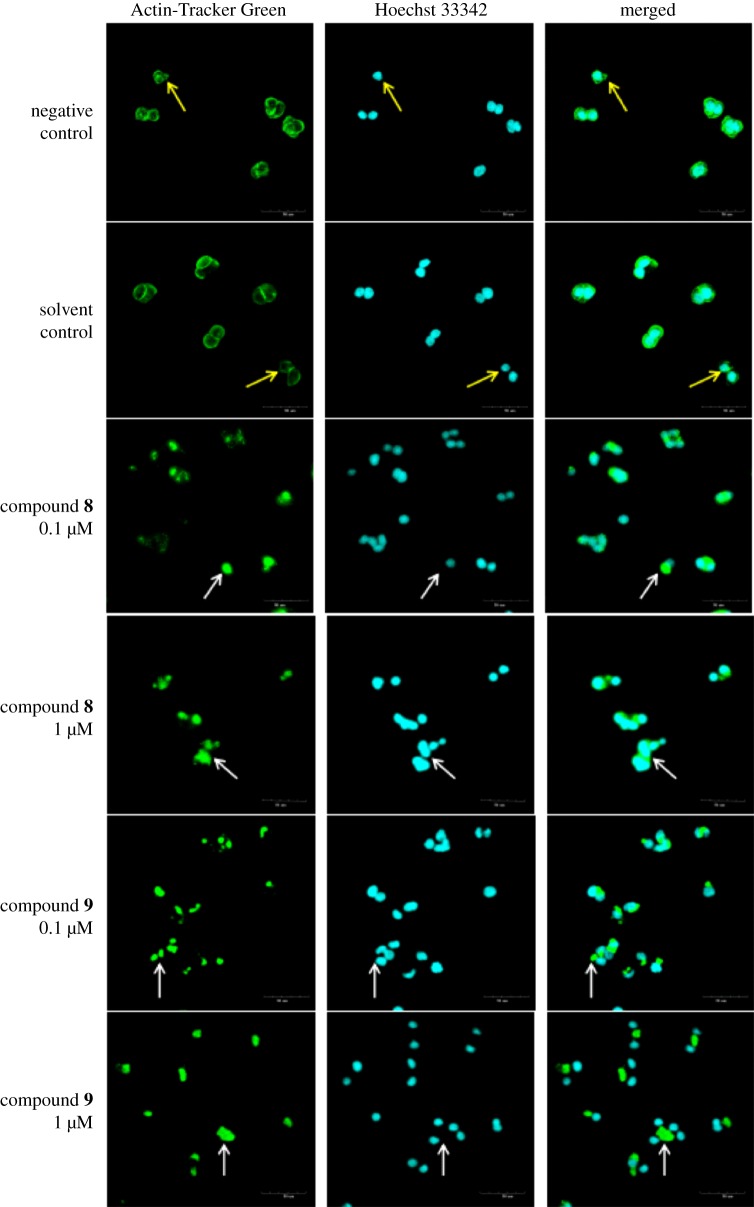
16,25-*O*-diacetyl-cucurbitane F (**8**) and 25-*O*-acetyl-23,24-dihydrocucurbitacin F (**9**) induced the morphological changes in the HT29 cells. Fluorescence photomicrographic images of HT29 cells stained with Actin-Tracker Green (actin, green) fluorescent probe and Hoechst 33342 (DNA, blue). The HT29 cells were incubated with either DMSO (2‰), compound **8** (0.1, 1 µM, respectively) or compound **9** (0.1, 1 µM, respectively) for 24 h. The negative control was without treatment. Scale bar, 50 µm.

As the cytoskeleton is very important for maintaining the native morphology and function of cells, we investigated that the microfilaments change by staining F-actin after the treatment with compounds **8** and **9**. Negative control HT29 cells exhibited F-actin that mainly surrounded the edge of cells, especially in the contact area of cells (figure 6). Meanwhile, the formation of lamellipodia was observed at the leading edge (indicated by yellow arrows in figure 6). The microfilaments in solvent control cells (2‰ DMSO) were as intact as that of negative control, whereas destruction of cytoskeleton was observed in cells treated with compounds **8** and **9**. The distribution of F-actin was diminished significantly in area near the surface of the cells (figure 6). Overall, the F-actin aggregation (indicated by white arrows in figure 6) was increased with increasing concentration of compounds **8** and **9**. Aggregated F-actin completely detached from the nucleus after 24 h treatment with 1 µM compound **9** (figure 6). The results indicated that compound **9** is more potent than compound **8** in inducing F-actin aggregation.

## 4. Conclusion

^13^C NMR data play an important role in inferring the structure of triterpenoids. Based on previous reports and our data in this paper, the ^13^C NMR spectral characteristics of common substituents of 5,24-diene cucurbitane tetracyclic triterpenes were summarized [4–8,11–13]. In the A ring, with a broad singlet signal (H-3) in ^1^H NMR, the 3β-OH was often connected with sugar, the obvious characteristic is that the *δ*_C-3_ 75–81 (CH) was replaced by 85–90 (CH). In the B ring, the presence of *δ*_C_ 140–146 (C-5, C) and 116–122 (C-6, CH) indicated that there exists a double bond. In the C ring, the big changes often occur at *δ*_C-11_ 211–219 (C, carbonyl), 77–80 (CH, *α*-OH) with 1H (dd, *J* = 10–12, 4–6 Hz), 72–74 (CH, *β*-OH) with 1H (dd, *J* = 3–5, 1–3 Hz) and 26–32 (CH_2_). In the side chain, it was *δ*_C-20_ 73–82 (C) instead of 35–38 (CH) that revealed the appearance of OH (C-20). In addition, the substituents of terminal Δ^24^, such as *δ*_C_ 64–68 (C-26, CH_2_OH), 58–62 (C-27, CH_2_OH), 21–23 (C-26, CH_3_) and 13–15 (C-27, CH_3_), have been identified. These data could help the interpreter to infer the characteristic groups quickly.

We compared the structures of cucurbitane triterpenoids with anti-cancer activity, which could provide direction for structural modification of triterpenoids. Cucurbitacins with carbonyl group at C-11, such as cucurbitacin B, D, E, I and compounds **8** and **9**, exhibited strong anti-cancer activity. The α-hydroxy-ketone at C-21 and C-22 might be another important active group. And it could be deduced that a hydroxyl at C-16 would enhance cytotoxicity from the comparison results of compounds **8** and **9** in this paper.

Moreover, we not only investigated the effects of compounds **1**–**9** on HT29 cell proliferation, but also preliminarily explored the mechanism of compounds **8** and **9** on anti-proliferative effect in colon cancer cells. Cucurbitacins are a group of highly oxidized tetracyclic triterpenoids, and the screening study of these on the cytotoxic activity indicated that cucurbitacins possess strong anti-cancer activity, especially cucurbitacin B, D, E, I and compound **9** [3,10,14–17]. The report pointed out that cucurbitacin-I induced G_2_/M cell cycle arrest in SW480 cells accompanied by the downregulation of cyclin A, cyclin B1, CDK1 and CDC25C *in vitro* and *in vivo* [18]. However, cucurbitacin B induces cell cycle G_2_ arrest in SW480 cells without downregulation of CDK1 expression [19]. Besides cell cycle arrest, apoptosis is another mechanism for anti-proliferative effect of cancer cell. Cucurbitacin-I induced apoptosis in SW480 cells via downregulation of Bcl-2 expression and increasing the expression of apoptosis-related proteins (cleaved caspases-3, -7, -8 and -9, and PARP) [18]. But cucurbitacin B-induced apoptosis did not alter the expression of anti-apoptotic proteins such as Bcl-2 and Bcl-xL [19]. Thus, there are controversies about the anti-cancer mechanisms of different cucurbitacins even for the same cells. Certainly, there are also differences in anti-tumour mechanisms of same cucurbitacin on different cell lines [20,21]. In addition, recent studies have reported that cucurbitacins have an inhibitory effect on the proliferation of cancer cells, as well as on the polymerization and permeability of actin [22–24]. Therefore, we need further investigation to clarify their mechanisms.

In conclusion, our studies enriched the chemical composition research of *H. pengxianensis* and provided scientific basis for the development of 16,25-*O*-diacetyl-cucurbitane F (**8**) and 25-*O*-acetyl-23,24-dihydrocucurbitacin F (**9**) as a chemotherapeutic agent against colon cancer.

## Data accessibility

This article does not contain any additional data.
